# Who Is He? Children with ASD and ADHD Take the Listener into Account in Their Production of Ambiguous Pronouns

**DOI:** 10.1371/journal.pone.0132408

**Published:** 2015-07-06

**Authors:** Sanne J. M. Kuijper, Catharina A. Hartman, Petra Hendriks

**Affiliations:** 1 Center for Language and Cognition Groningen, University of Groningen, Groningen, The Netherlands; 2 Interdisciplinary Center Psychopathology Emotion regulation (ICPE), University Medical Center Groningen, Groningen, The Netherlands; University of Wisconsin-Madison, UNITED STATES

## Abstract

During conversation, speakers constantly make choices about how specific they wish to be in their use of referring expressions. In the present study we investigate whether speakers take the listener into account or whether they base their referential choices solely on their own representation of the discourse. We do this by examining the cognitive mechanisms that underlie the choice of referring expression at different discourse moments. Furthermore, we provide insights into how children with Autism Spectrum Disorder (ASD) and Attention Deficit Hyperactivity Disorder (ADHD) use referring expressions and whether their use differs from that of typically developing (TD) children. Children between 6 and 12 years old (ASD: n=46; ADHD: n=37; TD: n=38) were tested on their production of referring expressions and on Theory of Mind, response inhibition and working memory. We found support for the view that speakers take the listener into account when choosing a referring expression: Theory of Mind was related to referential choice only at those moments when speakers could not solely base their choice on their own discourse representation to be understood. Working memory appeared to be involved in keeping track of the different referents in the discourse. Furthermore, we found that TD children as well as children with ASD and children with ADHD took the listener into account in their choice of referring expression. In addition, children with ADHD were less specific than TD children in contexts with more than one referent. The previously observed problems with referential choice in children with ASD may lie in difficulties in keeping track of longer and more complex discourses, rather than in problems with taking into account the listener.

## Introduction

One of the most important functions of language is reference. When people communicate, they refer to the world around them. To refer to a particular referent, speakers have to choose how specific they wish to be in their use of a referring expression. For instance, to refer to Rembrandt van Rijn a speaker may use a specific expression, such as ‘the famous Dutch painter’, or a less specific referring expression, such as the pronoun ‘he’. The less specific a referring expression is, the more ambiguous the expression usually is. Pronouns in particular can have a wide range of possible interpretations and thus can be misunderstood by the listener. For example, in a context with two referents, the use of a pronoun may lead to an incorrect interpretation: ‘Rembrandt van Rijn had a different painting style than Vincent van Gogh. He painted “The three singers”.’ Here, the pronoun ‘he’ could be interpreted as referring to the intended referent but also to the other referent. In contrast, in a context with only one referent, the use of a pronoun is likely to lead to a correct interpretation: ‘Rembrandt van Rijn is a famous Dutch painter. He painted “The Night Watch”.’

Adult speakers tend to use referring expressions that are unambiguous with respect to the discourse (e.g., [1,2]). They generally avoid pronouns in situations in which the use of a pronoun may result in a non-intended interpretation. In contrast, young typically developing (TD) children often show difficulties with the appropriate choice of referring expression. Up to the age of ten, they tend to overuse pronouns, leading to ambiguity for the listener [2–5]. Problems with the correct use of referring expressions have also been found in older children with Autism Spectrum Disorder (ASD) or Attention Deficit Hyperactivity Disorder (ADHD), two neurodevelopmental disorders [6,7]. Why do young TD children and children with neurodevelopmental disorders have difficulties in their choice of referring expression, and, more generally, which linguistic and cognitive skills are needed to come to a correct decision about which form to use?

### Linguistic approaches to the choice of referring expressions

Two linguistic approaches to modeling the choice of referring expressions have been proposed: the discourse-based approach (e.g., [8]) and the listener-based approach [4,9,10]. The discourse-based approach assumes that speakers base their choices on the properties of the discourse. Referents that are highly accessible in the linguistic discourse are referred to with less specific forms, while less accessible referents are referred to with more specific forms. Accessibility is thought to depend on givenness (whether a referent is new or already referred to earlier in the discourse; e.g., [11]), recency (how long ago the referent was mentioned before in discourse; e.g. [12,13]), syntactic prominence (the grammatical function of the referent in the previous utterance; e.g., [1]) and first mention (which referent was mentioned first in the discourse; e.g., [14]). Speakers use pronouns more often for given referents (high accessibility) than for new referents (low accessibility). Likewise, speakers tend to use a pronoun more often when a referent has recently been mentioned, when referring to characters in subject position, and when a referent is the first-mentioned referent. When the referent is less accessible, more specific forms such as full noun phrases (NPs) are used. Thus, according to the discourse-based approach, the speaker chooses a referential form on the basis of the accessibility of the referent in the discourse and does not take the listener into account. When encountering a referring expression, the listener has to ‘do the work’: he needs to infer the intended referent by comparing the discourse accessibility of the available referents with the degree of specificity of the referential form the speaker used.

In contrast to the discourse-based approach, the listener-based approach assumes that a speaker additionally takes into account the listener’s perspective [4,9,10]. According to this view, the speaker’s choice is influenced by assumptions about the listener’s knowledge and focus of attention. Speakers need to estimate how the form they use will be interpreted by the listener. This can be explained in pragmatic terms or in grammatical terms. The pragmatic account [10] is in accordance with Grice’s Maxim of Quantity, which states that speakers should make their contribution as informative as required, but not more informative than needed [15]. Alternatively, the speaker’s estimation of the listener’s interpretation can also be explained in grammatical terms, as in Hendriks, Englert, Wubs and Hoeks’ [16] optimality theoretic account of referential choice. These authors state that speakers prefer to use pronouns, but take into account the grammatical perspective of the listener to check whether the listener will be able to infer the correct referent. If not, (adult) speakers will use a more explicit form, such as a full NP. Thus, in the pragmatic as well as the grammatical account, speakers are assumed to have a preference for using short and therefore less informative expressions. This only results in communicative success if they take into account the listener, who requires expressions that are sufficiently specific to be able to identify the intended referent.

Previous studies yielded mixed results regarding the question whether speakers consider the listener’s perspective. Some studies found that speakers provide more information when the intended referent may be hard to identify for the listener, such as when several referents are present in the discourse [17]. In such situations, speakers use more explicit referential expressions than when only one referent is present, suggesting that they take into account their listeners. In contrast, other studies found that speakers sometimes do not provide as much information as they could. For example, speakers do not consistently avoid temporary ambiguities in their syntactic choices [18,19], thus causing difficulties in interpretation for their listeners. This seems to indicate that speakers do not consistently take into account their listeners. On the basis of these mixed results about speakers’ consideration of the listener’s perspective, we propose that speakers can sometimes rely on the discourse alone in their referential choice; however, in other situations, such as when the speaker’s focus of attention switches to another referent, they may need to take into account the listener and explicitly signal this shift in focus. It should be noted that in many situations it is difficult to decide whether the choice for a less or more specific referential form is driven by the discourse alone or also by the listener’s needs, since speakers and listeners are often present in the same discourse context and therefore often have the same focus of attention. The two explanations for speakers’ referential choices can only be teased apart using a reference production task that is designed in such a way that a distinction can be made between moments at which a speaker can merely rely on the discourse and other moments at which a speaker needs to consider the listener’s perspective as well (cf. [4]).

### Cognitive mechanisms

In the present study we investigate different mechanisms that may underlie speakers’ discourse-oriented and listener-oriented choices. It has been suggested that for a correct representation of the discourse sufficient working memory capacity is needed [20,21]. For listener-oriented choices, Theory of Mind processes are expected to be necessary [7,9]. Furthermore, inhibition may be needed, since speakers must block the form that is optimal from the speaker’s perspective in order to produce the form that is optimal for the listener (cf. [22]). Although previous studies suggested that these cognitive mechanisms may be involved, the direct relation between these mechanisms and the choice of referring expression has not been investigated so far. The relation between the choice of referring expressions and Theory of Mind, inhibition and working memory at different moments in the discourse can give us insights in whether and when speakers take into account their listeners.

If difficulties with the correct use of referring expressions are due to problems with taking into account the listener’s perspective or keeping track of accessibility of the referents in the linguistic discourse, people who have deficits in Theory of Mind, inhibition, or working memory are expected to show difficulties in the appropriate choice of referring expression. Children with ASD are known for difficulties with Theory of Mind (e.g., [23]); in children with ADHD, problems with Theory of Mind have also been reported [24], although not consistently [25,26]. Furthermore, working memory problems and problems with inhibition have been reported in children with ADHD and children with ASD (e.g., [27–30]). By including children with ASD and ADHD in our study, we attempt to maximize the variation in performance regarding these cognitive mechanisms and relate this variation to the choice between potentially ambiguous pronouns and unambiguous full NPs at various moments in the discourse.

### Reference in ADHD and ASD

Regarding children with ADHD, little if anything is known about their production of referring expressions. One study has shown that children with ADHD use more ambiguous referring expressions than TD children [6].

Studies on the choice of referring expressions of individuals with ASD thus far have shown mixed results: overspecification (use of full NPs in situations in which pronouns would suit) [7,31], underspecification (use of pronouns in situations in which this would lead to unclear or unintended reference) [31–33] and the choice of similar forms as in TD children [7,34] have all been reported. This variation in results may be partly caused by the fact that most studies have used narratives based on wordless picture books [31,32,34] or cartoons [7]. Although these narratives are more structured than free conversation in, for example, a play setting (such as in [33]), they still have the disadvantage that the stories are relatively long and eventful. Both children with ASD and children with ADHD have difficulties in producing coherent narratives [34–36]. Since the choice of referring expression highly depends on the previous discourse, this may cause difficulties in comparing the narratives of these children to the narratives of TD children and consequently in comparing their choices of referring expression. Hence, it is important to keep the discourse as similar as possible across children.

### Methodological approach and hypotheses

In narratives (such as in [7,31,32,34]), it is hard to determine the exact moments at which speakers may need to take into account their listeners. In contrast, the design of the present study allows for a detailed investigation of the speaker’s ability to take into account their listeners at different moments in the discourse. Three moments are distinguished: 1) Introduction of new referents; 2) Maintenance of reference; and 3) Reintroduction of a previously mentioned referent that is not the discourse topic at that moment (we use the term discourse topic to refer to the most prominent referent at a certain moment in the discourse). The choice of referring expression is expected to vary during these three particular points in discourse. Furthermore, we expect that the need to take the listener into account also varies across these discourse moments. Consequently we predict that Theory of Mind, inhibition and working memory have different effects on referential choice at these three discourse moments.

At the first discourse moment, when new referents are introduced, generally full NPs are used. Since the referents are new in discourse, no previous activation is expected. Given that sufficient working memory is only needed to keep referents activated and hence accessible [20], we hypothesize that working memory is not related to the choice of referring expression to introduce new characters. Furthermore, for the introduction of new referents speakers do not need to consider the listener’s knowledge, but can rely solely on the discourse. Therefore we do not expect associations between the choice of referring expression and Theory of Mind or inhibition. In addition, children with ASD and children with ADHD are not expected to have difficulties with the appropriate choice of referential expressions at the introduction moments, as it is not necessary at this moment in the discourse to take the other person's perspective or to keep referents activated.

At the second discourse moment, when maintaining reference to a character, it is expected that less specific forms are used. These may be pronouns if the character is highly prominent. However, when two characters are present in the discourse, more full NPs are expected to be used to maintain reference compared to when only one character is present [4,17]. For maintaining reference, the speaker only needs to consider the previous discourse without taking into account the listener’s knowledge. Consequently, we expect working memory but not Theory of Mind to be related to the choice of referring expression during the maintenance of reference in the discourse.

At the third discourse moment, when a speaker reintroduces a character that has been mentioned before but is no longer the topic of the discourse, the situation is different. Speakers cannot rely solely on the discourse and use the more economical pronoun, but need to take into account the listener’s focus of attention. If a speaker were to use a pronoun, this would be interpreted as referring to the current topic instead of to the character that is reintroduced. Therefore, to reintroduce a character after a topic shift to another character, speakers need to take the listener into account. We therefore predict Theory of Mind and inhibition to be associated with the choice of referring expression when reintroducing a referent. Working memory is also predicted to be associated with the choice of referring expressions, since speakers should keep track of the accessibility of the discourse referents.

Our experiment is set up in such a way that we can predict from the properties of the discourse when speakers must consider the needs of the listener. The stories are designed in such a way that a narrative structure is elicited that includes the three discourse moments discussed above: the stories start with only one character; halfway through the story a topic shift is elicited from this first character to a second character; at the end of the story a second topic shift is elicited from this second character back to the first character. Previous studies using these story books showed that this narrative structure could be successfully elicited in children from age 4 on [4,16]. Because of their problems with Theory of Mind, inhibition and working memory, we expect children with ASD and children with ADHD to show difficulties with the appropriate choice of referring expression when maintaining reference and when reintroducing a referent after a topic shift. The present study will provide a detailed examination of the choice of referring expression and the relation with cognitive factors in children with ASD or ADHD and TD children. This study is the first to investigate the choice of referring expression in TD children, children with ASD and children with ADHD at a detailed level, allowing us to distinguish between discourse-based and listener-based choices.

## Materials and Methods

### Participants

In total, 126 children were tested (51 with ASD, 37 with ADHD and 38 TD children), ranging in age from 6;1 to 12;10 (M = 9;1, SD = 1;9).

#### ASD

Children in the ASD group were diagnosed with Autistic Disorder (N = 10), PDD-NOS (N = 34) or Asperger’s Disorder (N = 7) by clinicians on the basis of the DSM-IV-TR criteria [37]. Then, both the Autism Diagnostic Interview Revised (ADI-R; [38]) and the Autism Diagnostic Observation Schedule (ADOS; [39]) were administered by certified psychologists. Children in this study were included in the ASD group if they met ADOS criteria for autism or ASD and/or ADI-R criteria for autism or ASD (cf. Risi et al.'s ASD2 criteria [40]). Of all children who were diagnosed with ASD, 29 children met the criteria on both instruments, 14 children only met the criteria on the ADI-R and 5 only met the criteria on the ADOS. Three children from the ASD group were excluded from further analysis because they did not meet the criteria on either instrument, leaving 48 children in the ASD group.

#### ADHD

Children in the ADHD group were diagnosed with Combined type (N = 19), Predominantly Hyperactive-Impulsive type (N = 12) or Predominantly Inattentive type (N = 6) by clinicians on the basis of the DSM-IV criteria [37]. Furthermore, both the Parent Interview for Child Symptoms (PICS; [41]) and the Teacher Telephone Interview-IV (TTI-IV; [42]) were administered by trained clinicians. Six children with ADHD lacked TTI information. Four of them already scored above the cut-off for ADHD based on parent information alone. The remaining two children scored 1 point below the cut-off for ADHD. Since these children scored comparably on the PICS to the other children in the ADHD group (for whom TTI scores combined with their PICS scores reached the cut-off), we included them in the analyses. Children were recruited between diagnosis and their appointment with the psychiatrist to consider the potential start of medication use. Therefore, all children were free of psychostimulant medication at the day of testing.

#### TD

Children in the TD group had not been diagnosed with ASD or ADHD. Furthermore they did not have any history of neurological or psychiatric problems. The ADOS, ADI-R and PICS were administered by trained clinicians in this group as well. None of the children scored above the cut-offs for ASD or ADHD described above.

### Materials

#### Reference production task

**Fig 1:**
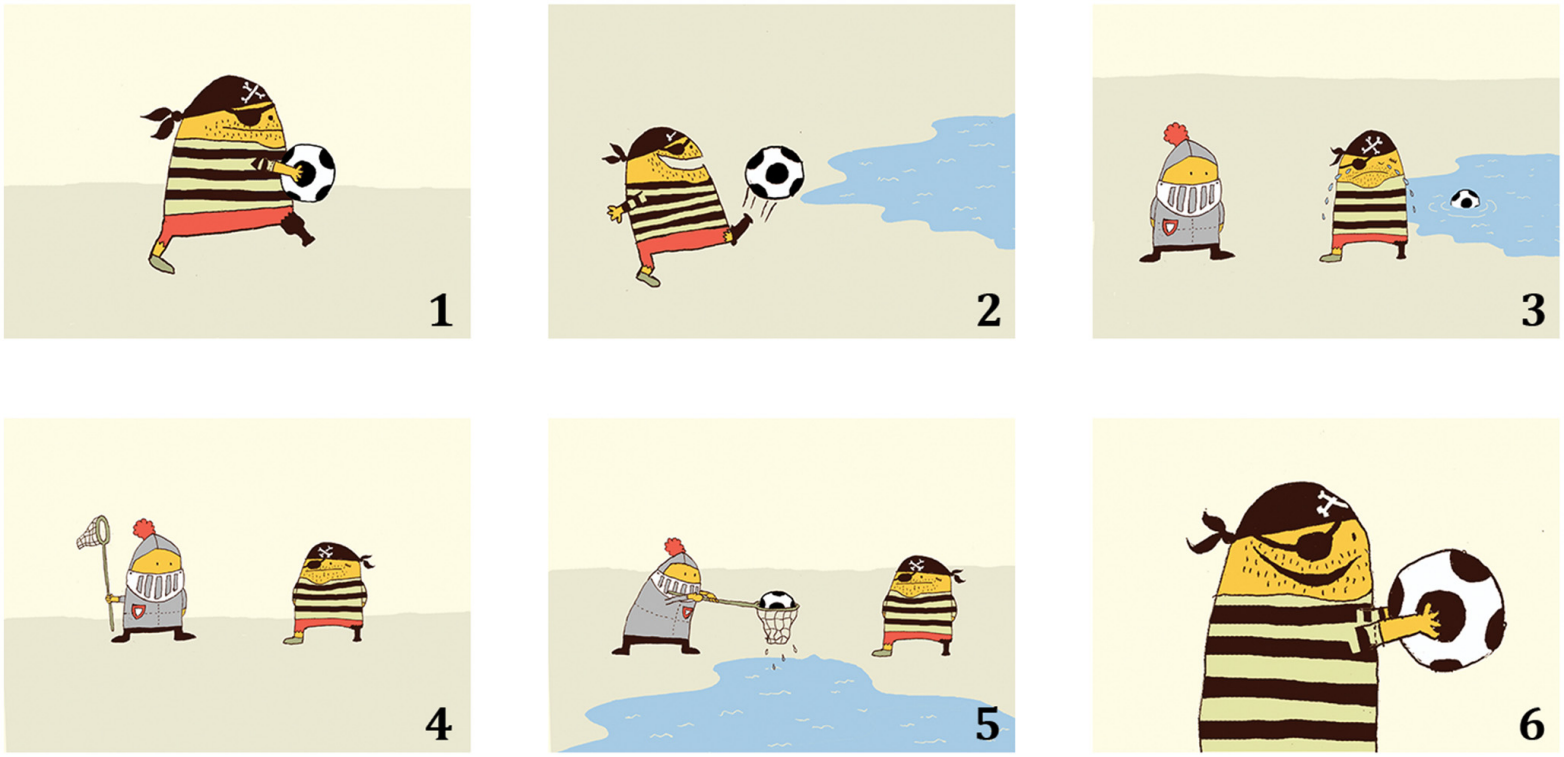
Example of storybook. Example of a storybook of the Reference Production Task. Each picture was shown on a separate page.

We used the reference production task from Hendriks et al. [4] that consisted of four storybooks containing six pictures each (see [43] for examples of the materials). Each picture was on a separate page. The four storybooks were constructed in the same way (Fig 1): in the first two pictures, one character was present, to prompt the introduction of a new character and the maintained reference to this character. We expected participants to introduce this character with a full NP (e.g., ‘the pirate’ or ‘a pirate’). To maintain reference to this character, we expected participants to mainly use pronouns. In the third picture a second character entered the story. In the fourth and fifth pictures, this character performed an action, in order to prompt the introduction of and maintained reference to the second character and thus a topic shift from the first to the second character. We expected that the second character would be introduced with a full NP. We expected participants to use either a pronoun or a full NP to maintain reference to this second character [4,17]. In the final picture, only the first character was present, to elicit the reintroduction of this character. We expected children who are able to take into account the listener’s perspective to reintroduce this character with a full NP. Contrastively, we expected children who have difficulties with taking into account another person’s perspective to refer to this character with a pronoun. In each story the two characters had the same gender. To ensure that the gender of the characters was clear to the children, we chose characters with stereotypical gender roles (e.g., fairy, princess, knight and pirate).

During the reference production task one experimenter was sitting next to the child to explain the task and turn the pages of the storybooks. The second experimenter was sitting further away and could not see the pictures. The production task started with an introductory page showing all the characters of the stories. Participants were asked to name all characters. If a participant did not know a character, the experimenter told him/her the right name and checked at the end whether the participant remembered. After the introductory page, the experimenter told a two-picture story as an example. Next, the participant was asked to tell a two-picture practice story. The first experimenter made clear to the participant that the second experimenter could not see the pictures and that the child had to make sure that the second experimenter also could understand the stories. After the practice session, the child was reminded that the second experimenter could not see the stories. Then the test session with the four stories started. The order of the test stories was reversed for half of the participants.

#### Theory of Mind

To test Theory of Mind, we used a task adapted from Hollebrandse, Van Hout and Hendriks [44]. This task measured both first-order False Belief (in which the belief of one other person is involved) and second-order False Belief (which is the understanding of the beliefs of another person about a third person). The task consisted of eight stories, each of which was accompanied by four pictures that were presented one by one on a computer screen. The task was divided in two blocks with a short break in between. The order of stories was counterbalanced across participants. Each story contained one second-order FB question and two first-order FB questions. The first first-order FB question was asked in the middle of the story. The second first-order FB question was asked at the end of the story, preceded by the second-order FB question. The second first-order question was presented in order to check whether children had difficulties with the length and complexity of the story.

One item was removed from further analysis since item analysis showed that the response on this item differed from the other seven items: on the second first-order FB question, mean accuracy on this item was only .48, while mean accuracy on the other items varied between .79 and .92. Additionally, on this item, mean accuracy on the second-order FB question was higher (.80) than on the easier first-order FB question (.48). Inspection of this item revealed that its content differed from the other items in that an extra belief had inadvertently been introduced, which made the first-order FB answer less plausible.

Two dependent measures were calculated: mean accuracy on the first first-order FB question (FB1) and mean accuracy on the second-order FB question (FB2).

#### Response inhibition

Response inhibition was tested with a stop-signal task adopted from Van den Wildenberg and Christoffels [45]. In this task, simple drawings of a tree and a door were presented on the computer screen. During go trials, participants were asked to press the button corresponding with the picture on a two-button box. In 30% of the trials, a visual stop signal was presented: a red square frame surrounding the picture border. When confronted with the stop signal, participants had to inhibit the go response by not pressing the button. The interval between the onset of the go picture and the onset of the stop signal (stop-signal delay) was set at 200 ms in the first stop trial. An online tracking algorithm adjusted stop-signal delay as a function of individual stopping performance [46]. If the participant was able to stop, the stop-signal delay increased by 50 ms, thereby decreasing the chances of successful inhibition in the next stop-trial. After a failed-inhibition trial, the stop-signal delay decreased by 50 ms. This adaptive algorithm ensured successful inhibition in about 50% of the stop trials, a procedure that yields reliable estimates of the Stop Signal Reaction Time (SSRT; [47]). SSRT was calculated as measure for response inhibition.

#### Working memory

Working memory was tested with an n-back task including three experimental conditions: 0-back (baseline), 1-back and 2-back. In each condition, pictures were presented randomly on a computer screen with stimulus duration of 1000 milliseconds, followed by an interstimulus interval of 1500 milliseconds. In the 0-back condition, participants were instructed to press the yes-button when they saw a picture of a car, and to press the no-button when another picture appeared. In the 1-back condition, participants had to press the yes-button when the picture matched the picture immediately preceding it, and otherwise press the no-button. In the 2-back condition, participants had to press the yes-button when the picture matched the picture that appeared two pictures back. Participants started with a practice session of 15 trials per condition (0-, 1- and 2-back), followed by a test session consisting of four sequences of 15 trials per condition (resulting in 60 trials per condition). The total numbers correct on the 2-back condition was calculated as measure of working memory (WM).

#### Background variables

IQ was assessed by two subtests (Vocabulary and Block Design) of the Dutch Wechsler Intelligence Scale for Children (WISC-III NL; [48]). Verbal ability was assessed by the Peabody Picture Vocabulary Test-III-NL (PPVT; [49]). In Table 1 the mean scores per participant group on the background variables are presented, as well as the mean scores on the diagnostic instruments and the Theory of Mind, response inhibition and working memory tasks.

**Table 1:** Mean Scores (Standard Deviations) of age, clinical interviews, WISC-III, PPVT, Theory of Mind task, n-back task, Stop task per Participant Group.

	ASD (*n* = 46)^a^		ADHD (*n* = 37)^a^		TD (*n* = 38)^a^		Group differences (Bonferroni corrected post hoc analyses)
	M	(SD)	M	(SD)	M	(SD)	
**% Male**	87		84		66		
**Age**	9;3	(1;10)	8;9	(1;7)	9;0	(1;9)	n.s.
**ADI-R** ^b^ **Social Interaction**	16.46	(6.19)	4.51	(4.07)	1.82	(3.09)	ASD***>ADHD>TD*
**ADI-R** ^b^ **Communication**	12.72	(4.57)	3.97	(2.66)	1.42	(1.80)	ASD***>ADHD>TD**
**ADI-R** ^b^ **Stereotyped Behavior**	4.39	(2.56)	1.41	(1.53)	0.32	(0.66)	ASD***>ADHD>TD*
**ADI-R** ^b^ **Behavior < 3 yrs**.	2.98	(0.98)	1.49	(1.52)	0.13	(0.41)	ASD***>ADHD>TD***
**ADOS** ^c^ **Communication**	2.78	(1.51)	1.06	(0.92)	0.53	(0.76)	ASD***>ADHD, TD
**ADOS** ^c^ **Social interaction**	7.44	(3.22)	2.67	(2.01)	1.50	(1.72)	ASD***>ADHD, TD
**ADOS** ^c^ **Com+Soc**	10.22	(4.36)	3.72	(2.56)	2.03	(1.99)	ASD***>ADHD, TD
**ADOS RRB**	1.22	(1.36)	0.28	(0.57)	0.16	(0.44)	ASD***>ADHD, TD
**ADOS Social Affect**	9.09	(4.37)	2.94	(2.43)	1.74	(2.02)	ASD***>ADHD, TD
**ADOS SA+RRB**	10.31	(5.00)	3.22	(2.43)	1.89	(2.15)	ASD***>ADHD, TD
**PICS** ^d^ **Inattention**	2.35	(2.06)	3.54	(2.19)	0.11	(0.39)	ADHD**>ASD>TD***
**PICS** ^d^ **Hyperactivity/impulsivity**	2.04	(1.99)	5.11	(2.51)	0.29	(0.57)	ADHD***>ASD>TD***
**WISC-III Block Design**	9.85	(3.68)	8.35	(2.98)	11.16	(3.23)	ADHD<TD**
**WISC-III Vocabulary**	8.65	(3.34)	9.49	(2.09)	11.82	(2.51)	ASD***, ADHD**<TD
**WISC-III Estimated IQ**	95.45	(18.73)	93.44	(12.67)	109.02	(13.64)	ASD, ADHD<TD***
**PPVT WBQ**	104.09	(15.27)	100.22	(12.49)	108.84	(10.72)	ADHD<TD*
**Theory of Mind Task (Proportion correct FB1)**	0.87	(0.21)	0.87	(0.14)	0.94	(0.11)	n.s.
**Theory of Mind Task (Proportion correct FB2)**	0.55	(0.40)	0.56	(0.34)	0.78	(0.29)	ASD, ADHD<TD*
**N-Back Task (Numbers correct 2-back)**	38.95	(8.13)	38.19	(7.45)	41.77	(5.28)	n.s.
**Stop-Signal Task (SSRT)**	259.64	(98.09)	254.84	(94.25)	256.74	(77.59)	n.s.

* = *p* < .05;

** = *p* < .01;

*** = *p* < .001;

n.s. = non-significant.

^a^ Number of participants may vary per task, since some children did not finish all tasks (see Procedure).

^b^ Five children in the ADHD group scored on the ADI-R above the cut-off for ASD (on the basis of Risi et al.’s criteria [40]).

^c^ Two children in the ADHD group scored above the ADOS criteria for ASD.

^d^ Seven children in the ASD group scored within our criteria for ADHD on the PICS (above or one point below the cut-off on the PICS).

### Procedure

Children and their parents were recruited by brochures at schools and in outpatient clinics for child and adolescent psychiatry in Groningen. They took part in a larger study on communication in ASD and ADHD. The research protocol was evaluated by the Central Committee on Research Involving Human Subjects (CCMO) as not falling under the Medical Research Involving Human Subjects Act (WMO). Nevertheless, we followed the same procedures and obtained written informed consent of all parents for participation of their children. Children and parents came to the lab together. Children were tested individually on a single day in a quiet testing room with two experimenters present. Half of the children first performed the response inhibition task, followed by the reference production task, the Theory of Mind task and the working memory task. The other half of the participants received the tasks in reversed order.

Due to problems with the voice recorder, stories of two participants (both in the ASD group) were not recorded. These children were excluded from further analysis. Furthermore, only two of the four stories of two children (1 ASD and 1 TD) and three out of four stories of one child (ADHD) were recorded. These stories were included in analyses, which led to 479 stories of 121 children in total. One child (ASD) conducted only half of the Theory of Mind task and was removed from further analyses including the Theory of Mind task. This child and three additional children (2 ASD, 1 ADHD) did not finish the working memory task and were removed from analyses that included this task. Three of these (2 ASD and 1 ADHD) and one additional child (ADHD) were not able to complete the inhibition task and consequently were excluded from analyses involving this task.

### Coding of production data

The narratives collected in the production task were voice-recorded. After the test sessions, the narratives were transcribed by an independent transcriber and checked by a second transcriber. Next, all narratives were coded for topic shifts. We used Hendriks et al.’s [4] step-wise instructions, which are based on the rules from Centering Theory [50,51]: (1) list all referring expressions in each utterance, (2) remove all referring expressions that were not referred to in the prior utterance, (3) if the resulting list contains exactly one pronoun, the referent of the pronoun is the topic, (4) if the resulting list contains no pronouns or more than one pronoun, the referent in the current utterance that was syntactically most prominent in the prior utterance is the topic. In step four, the prominence was based on grammatical function (‘subject>object(s)>other’) [51]. A topic shift was coded if the discourse topic at a certain point in the narrative differed from the previous topic. Two coders scored independently for presence of a topic shift. Inter-scorer agreement was high (Cohen’s kappa = .95). In 96% of all narratives (462 out of 479 stories) a topic shift was established.

Finally, in all narratives with a topic shift, five references to one of the two characters were coded for their grammatical form: the first reference to the first character (Intro-1); the next reference to the first character following an utterance with a reference to this character (Maintain-1); the first reference to the second character (Intro-2); the next reference to the second character following an utterance with a reference to this character (Maintain-2); and the first reference to the first character as subject of the sentence after the topic shift (Reintro-1). In 1.3% of all items, the child did not produce any of these five references. Two coders independently scored all references for their grammatical form (Cohen’s kappa = .98).

### Data analysis

The data were analyzed using Generalized Linear Mixed Models. The outcome variable (NP use) was coded binary (0 for the use of a pronoun and 1 for the use of a full NP). A logit link was used to accommodate this repeatedly measured binary outcome variable [52,53]. The covariance matrix of our dependent variable was in agreement with compound symmetry, which was used for analysis. We performed analyses separately for five different moments in discourse: Intro-1, Maintain-1, Intro-2, Maintain-2 and Reintro-1. First we tested for differences between groups in referential choice at these five moments. Contrasts between diagnostic groups and controls (ASD vs. TD and ADHD vs. TD) were dummy-coded and included as fixed factors in the analysis. Next we examined possible mechanisms underlying referential choice by including the relevant parameters derived from the Theory of Mind task (FB1 and FB2), the n-back task (WM) and the Stop-Signal task (SSRT), respectively. All four parameters were mean-centered around a value of zero and were included, in four separate analyses, as fixed factors in the aforementioned model. Interactions or Group effects that had no effect on Accuracy (p > .05) were removed from the model. Finally, we tested whether found associations held up when all main and interaction effects with a significance value of p ≤ .05 were examined simultaneously in a multiple GLMM analysis.

## Results

### Group effects

**Fig 2:**
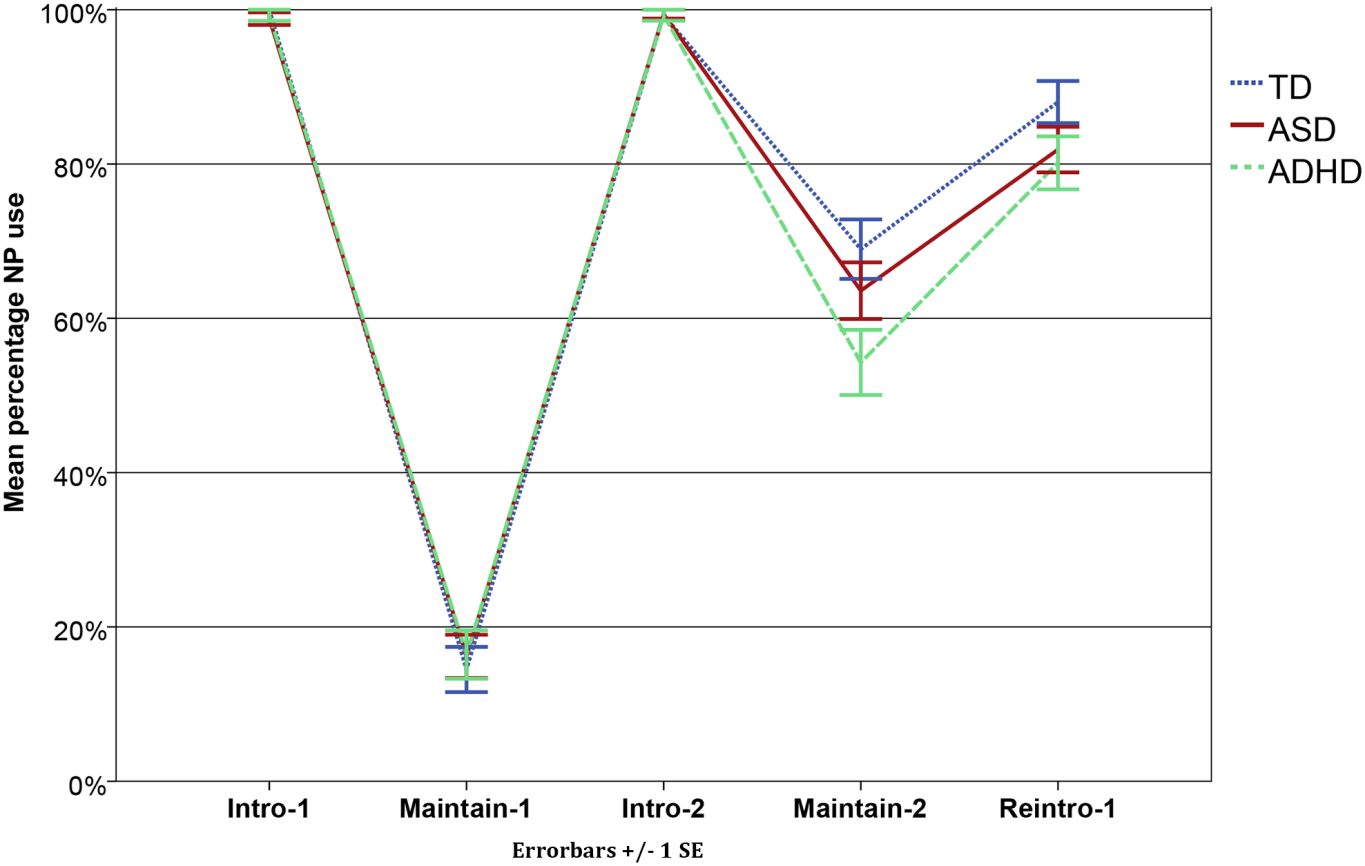
Mean percentages full NP use per Group and per Discourse Position. Mean percentage full NP use per Discourse Position for the TD group (blue dotted line), the ASD group (red solid line), and the ADHD group (green dotted line). Intro-1: Introduction of first character; Maintain-1: Maintenance of reference to first character; Intro-2: Introduction of second character; Maintain-2: Maintenance of reference to second character; Reintro-1: Reintroduction of first character. Error bars represent 1 SE.

The percentage of full NP use per group at the different positions in discourse is shown in Fig 2.

#### Introduction positions

We start with the referring expressions children use to introduce a new character (Intro-1 and Intro-2). As expected, no differences were found between the groups at these two reference positions (Intro-1: ASD vs. TD (*B* = -6.12, *SE* = 13.50, *p* = .65); ADHD vs. TD (*B* = -5.64, *SE* = 13.51, *p* = .68); Intro-2: ASD vs. TD (*B* = .19, *SE* = 1.43, *p* = .89); ADHD vs. TD (*B* = -.015, *SE* = 1.43, *p* = .99)). Children with ASD, children with ADHD and TD children all mainly use full NPs to introduce new characters.

#### Maintenance positions

When only one character is present in the story (Maintain-1), all three groups mainly use a pronoun to maintain reference to this character (Mean NP use: ASD: M = 15%; ADHD: M = 16%; TD: M = 14%; ASD vs. TD (*B* = .14, *SE* = .42, *p* = .75); ADHD vs. TD (*B* = .20, *SE* = .44, *p* = .65)). A different pattern arises when two characters are present in the story (Maintain-2): children with ADHD (M = 54%) produce fewer full NPs than TD children (M = 69%) when they maintain reference to the second character (*B* = -.63, *SE* = .26, *p* = .015). Children with ASD (M = 64%) do not significantly differ from the TD group (*B* = -.24, *SE* = .25, *p* = .34).

#### Reintroduction position

To avoid misunderstanding, the reintroduction of the first character after a topic shift needs to be done by the use of a full NP. No group differences were found. In all three groups, children use a full NP in most cases (ASD: M = 82%; ADHD: M = 80%; TD: M = 88%; ASD vs. TD (*B* = -.49, *SE* = .39, *p* = .22); ADHD vs. TD (*B* = -.60, *SE* = .41, *p* = .14)).

Next to the differences between groups in reference production, we tested which mechanisms may underlie the choice between a specific or less specific referring expression.

### Mechanisms

#### Introduction positions

All children use full NPs to introduce new referents (ASD and ADHD groups in 99% and TD group in 100% of the cases), leaving no variation to statistically test for mechanisms underlying referential choice in the introduction positions.

#### Maintenance positions

No significant associations with referential choice for Maintain-1 and any of the mechanisms were found (see Table 2). On the Maintain-2 position, an interaction effect of ADHD and working memory was found in addition to the (already identified) main effect of ADHD. To probe the interaction effect, we performed follow-up analyses per group. Again, we used Generalized Linear Mixed Models with logit link with NP use as binary coded outcome variable, and WM as fixed factor. Compound symmetry was used as covariance matrix. We did not find a significant effect of working memory in the TD group (*B* = -.05, *SE* = .04, *p* = .23), the ASD group (*B* = .03, *SE* = .02, *p* = .19), or the ADHD group (*B* = .05, *SE* = .03, *p* = .08). No interaction effects or main effects were found for the other mechanisms at Maintain-2.

**Table 2:** Estimated effects of Mechanism and Group on NP use per discourse position.

		FB1		FB2		SSRT		WM	
Position	Predictor	Estimate	SE	Estimate	SE	Estimate	SE	Estimate	SE
**Maintain-1**	**Mechanism**	-.28	1.03	-.34	.46	.002	.002	-.01	.02
**Maintain-2**	**Mechanism**	.60	.65	.17	.30	-.001	.001	-.05	.04
	**ASD vs. TD**	-.20	.26	-.20	.26	-.25	.25	-.34	.28
	**ADHD vs. TD**	-.59*	.26	-.59*	.27	-.68*	.26	-.66*	.28
	**Mechanism** * **ASD vs. TD**	-	-	-	-	-	-	.07	.04
	**Mechanism** * **ADHD vs. TD**	-	-	-	-	-	-	.10*	.05
**Reintro-1**	**Mechanism**	2.13**	.78	1.69***	.41	-.003*	.002	.08***	.02

* = *p* < .05;

** = *p* < .01;

*** = *p* < .001;

- = not included in final model.

### Reintroduction position

For reintroducing a referent, we found an effect of first-order Theory of Mind, second-order Theory of Mind, inhibition and working memory. No significant interaction effects or Group effects were found. In the multivariable analysis all mechanisms were included as fixed factors simultaneously. We found an effect of second-order Theory of Mind (*B* = 1.51, *SE* = .43, *p* < .001) and working memory (*B* = .06, *SE* = .02, *p* < .01), while first-order Theory of Mind and inhibition no longer were significantly associated with NP use.

## Discussion

### Speakers take the listener into account

Our first aim was to investigate the mechanisms underlying the choice of referring expression both at the moments at which a speaker can merely rely on the discourse and at the moments at which a speaker needs to consider the listener’s perspective. We hypothesized that working memory is involved in keeping track of the accessibility of the referents in the discourse, and thus is related to referential choice both when maintaining reference and when reintroducing referents. Furthermore we hypothesized that Theory of Mind and inhibition are related to the choice of referring expression only at the discourse moments at which speakers need to take the listener into account. We found support for these hypotheses: Theory of Mind performance, response inhibition (although the latter only in univariable but not multivariable analysis) and working memory are associated with the use of full NPs to reintroduce a character after a topic shift. The fact that inhibition is only associated with full NP use in univariable analysis suggests that Theory of Mind may be more important than inhibition in taking another person’s perspective.

The results of the present study are partly in line with a study on perspective taking in the interpretation of pronouns and reflexives in object position (‘the elephant is hitting *him/himself’*) by the same group of children as in the present study [22]. That interpretation study showed that listeners take into account the speaker’s grammatical perspective in the interpretation of pronouns. In the present study, we show that speakers also take into account the listener’s grammatical perspective in their choice of referring expression. Theory of Mind is associated with both the use of pronouns (namely, in the present study) and the interpretation of pronouns [22], suggesting that for linguistic reference one needs to consider the other perspective as well. As predicted, we only found an association between the choice of referring expression and Theory of Mind at the discourse moment of referent reintroduction, which was the only moment in our experimental set-up at which the speaker really needed to take the listener into account. However, this should not be taken to mean that speakers only take the listener into account at this moment. Rather, it is likely that speakers who are able to take the listener’s perspective into account do so constantly.

Working memory is associated with NP use at the moment of referent reintroduction. Furthermore, we found that working memory is also related to referential choice in the ADHD group at the point of maintained reference to the second character. At this moment in the discourse, the speaker does not necessarily have to take into account the listener’s perspective, but can rely on discourse alone. The association between working memory and referential choice in the ADHD group may reflect possible problems with keeping track of the discourse referents, rather than difficulty in taking into account the other person’s perspective. This is further confirmed by a comparison of the results of the present production study with Kuijper et al.’s (2014) interpretation study with the same children: in the present study, we find that working memory is associated with pronoun production, but it is not associated with pronoun interpretation in Kuijper et al.’s interpretation study [22]. In the present production study, a more extended discourse was available than in the interpretation study, in which the grammatical binding principles were investigated at the local level of the sentence. This may imply that working memory is more important in the representation of the discourse than in the consideration of the conversational partner’s grammatical perspective.

We do not find an association between working memory and referential choice in the ADHD group when participants had to maintain reference to the *first* character. At this moment, only one character is present. Hence, it may be that ADHD children with low working memory capacity are able to keep this single referent activated and thus highly accessible. Only when the discourse involves multiple referents, ADHD children with low working memory capacity may encounter problems in keeping track of these referents.

In sum, we found support for the listener-based account of referential choice. Theory of Mind and, to a lesser extent, inhibition are associated with the appropriate choice of referring expression at the discourse moment at which the speaker is expected to take into account the listener. Sufficient working memory may be needed to keep track of multiple referents in the discourse and select the appropriate referring expression based on their accessibility levels.

### Children with ASD and ADHD take the listener into account

The second aim of our study was to investigate the choice of referring expression in children with ASD and children with ADHD. We find that children with ASD or ADHD use the same referring expressions as TD children to introduce new referents and reintroduce a referent after a topic shift. In addition, children with ASD do not differ from TD children with regard to maintaining reference. By and large, then, children with ASD and ADHD use the same referring expressions at various discourse moments as TD children.

One divergence from this main conclusion is found. Although children with ADHD use the same expressions for introducing and reintroducing referents as the other children, they differ in the choice of referring expression when maintaining reference in a situation with two characters of the same gender. Previous studies have shown that in such situations adult speakers are more specific and more often use full NPs to maintain reference compared to situations with only one referent [4,17]. We also find this pattern in our study with children between the age of 6 and 12. However, children with ADHD more often use pronouns when maintaining reference to a second character than TD children. This use of less specific expressions has also been found in elderly adults (69–87 years old) and young TD children (4;3–6;5 years old; [4]).

In contrast to the children with ADHD in our study, the young TD children in the Hendriks et al. [4] study also used less specific expressions to reintroduce a referent. The children with ADHD in our study seem to resemble the elderly adults in the Hendriks et al. study [4] in their choice of referring expression: both groups produced less specific referring expressions to maintain reference to a second character, while using more specific referring expressions to reintroduce a referent, suggesting that they are able to take the listener into account. Hendriks et al. [4] suggested that the elderly adults had difficulty to determine the accessibility of the different referents and consequently resort to the more economical pronoun. Post-hoc investigation of the utterances preceding the utterance expressing maintained reference to the second character supports this suggestion. When children with ADHD referred to both characters in the preceding sentence, they use full NPs more often (in 77% of the cases) than when they referred to only one of the characters in the preceding utterance (47%). When both characters were just referred to, they may be equally activated, which promotes the use of the more specific full NP (cf. [4,17]). In addition, a recent study of reference production in a dual-task setting showed that increased cognitive load in adults led to an increase in pronoun use [21], confirming that insufficient working memory capacity leads to the use of less taxing referring expressions.

Our finding that children with ASD do not have problems in choosing the appropriate referring expression in a relatively controlled context such as in the present study does not imply that these children have no problems in other contexts. In particular, in previous studies that reported differences in referential choice in children with ASD compared to TD children, the discourse generally was longer and featured more characters. Furthermore, in our study children could see the pictures during storytelling. This contrasts with several other studies in which no visual support was available during narration (e.g., [7]), which may have placed a greater burden on working memory. Since children with ASD and ADHD show problems with coherent retelling of stories [34–36], it may be that in studies with long and complicated narratives these children lose track of the discourse and consequently fail to choose the appropriate referring expression. This is in line with our finding that the presence of a second character causes difficulty in referential choice for children with ADHD. As argued, this may be caused by difficulties in keeping track of the two referents and their accessibility. It could be that in less controlled discourses with more than two referents, as in previous studies, children with ASD also show difficulty in keeping track of the accessibility of the different referents. Therefore, although speculative, we propose that the difficulties found in previous studies may not be caused by problems in taking the listener into account, but rather by difficulties in keeping track of referents in a long and complex discourse.

It may be surprising at first glance that children with ASD and children with ADHD are able to take the listener into account in their choice of referring expression, given their known pragmatic difficulties. Hendriks et al. [4] propose in their Asymmetric Grammar Hypothesis that speakers model a hypothetical listener and listeners model a hypothetical speaker (thereby accounting for the coordination of referential choice between speakers and listeners). This hypothetical listener should be understood as the speaker imagining himself in the role of the listener. As this hypothetical listener has the same knowledge, beliefs and intentions as the speaker, this grammatical perspective taking may be less demanding than taking the perspective of an actual listener, who may have different knowledge, beliefs and intentions. Since this grammatical perspective taking does not vary per situation, it therefore could be automatized (cf. the computational simulations of Van Rij et al., [54]). The present results support this grammatical explanation of the listener-based account of referential choice. Children with ASD and children with ADHD may be able to take into account the grammatical perspective of a hypothetical listener. These findings are consistent with the findings from the interpretation study with the same group of children [22], suggesting that children with ASD and children with ADHD are capable of taking the speaker’s perspective as a listener. Nevertheless, these children may still show problems with considering the needs of an actual listener or speaker, who may not possess the same knowledge as the child itself. Additionally, it should be noted that in this study each child was explicitly told that the experimenter could not see the pictures. This is in contrast with daily conversation, in which a speaker must determine on his own the extent to which the listener has access to the same information as the speaker. Thus, first a speaker must determine which information is shared, and second he must choose the appropriate referential expression. This study showed that children with ASD and children with ADHD do not have problems with the second step. However, it could be that their problems in daily conversation are related to difficulties with realizing that the listener does not possess the same information.

In sum, we found that children with ASD and children with ADHD were able to take the listener into account in their choice of referring expression. Additionally, in discourses with only one referent, they were able to use the appropriate referring expression. Children with ADHD were less specific than TD children in contexts with more than one referent, which indicates that they may have difficulty with keeping multiple referents accessible in a more complex discourse.

### Conclusion

We found support for the listener-based account of referential choice and for the grammatical explanation of the coordination between speakers and listeners. We found that Theory of Mind and, to a lesser extent, inhibition were associated with the appropriate choice of referring expression at the discourse moment at which the listener’s perspective had to be taken into account. Sufficient working memory capacity appeared to be needed to keep track of the discourse referents and their accessibility. Furthermore, we found that children with ASD and children with ADHD took the listener into account in their choice of referring expression. Additionally, in a structured and simple discourse as in the present study, children with ASD and children with ADHD were able to use the appropriate referring expression. Their previously observed difficulties with the choice of referring expressions in discourse may lie with keeping track of longer and more complex discourses with many referents, rather than with taking into account the listener.
